# Characterizing human vestibular sensory epithelia for experimental studies: new hair bundles on old tissue and implications for therapeutic interventions in ageing

**DOI:** 10.1016/j.neurobiolaging.2015.02.013

**Published:** 2015-06

**Authors:** Ruth R. Taylor, Daniel J. Jagger, Shakeel R. Saeed, Patrick Axon, Neil Donnelly, James Tysome, David Moffatt, Richard Irving, Peter Monksfield, Chris Coulson, Simon R. Freeman, Simon K. Lloyd, Andrew Forge

**Affiliations:** a UCL Ear Institute, London, UK; b Royal National Throat Nose and Ear Hospital, UCLH NHS Foundation Trust, London, UK; c Addenbrooke's Hospital, Cambridge University NHS Foundation Trust, Cambridge, UK; d Queen Elizabeth Hospital, University Hospitals Birmingham NHS Foundation Trust, Queen Elizabeth Medical Centre, Birmingham, UK; e Manchester Royal Infirmary, Central Manchester University Hospitals NHS Trust, Manchester, UK; f Salford Royal Infirmary, Salford Royal NHS Foundation Trust, Salford, UK

**Keywords:** Human vestibular, Utricle, Hair cells, Supporting cells, Stereocilia, Aging pathologies

## Abstract

Balance disequilibrium is a significant contributor to falls in the elderly. The most common cause of balance dysfunction is loss of sensory cells from the vestibular sensory epithelia of the inner ear. However, inaccessibility of inner ear tissue in humans severely restricts possibilities for experimental manipulation to develop therapies to ameliorate this loss. We provide a structural and functional analysis of human vestibular sensory epithelia harvested at trans-labyrinthine surgery. We demonstrate the viability of the tissue and labeling with specific markers of hair cell function and of ion homeostasis in the epithelium. Samples obtained from the oldest patients revealed a significant loss of hair cells across the tissue surface, but we found immature hair bundles present in epithelia harvested from patients >60 years of age. These results suggest that the environment of the human vestibular sensory epithelium could be responsive to stimulation of developmental pathways to enhance hair cell regeneration, as has been demonstrated successfully in the vestibular organs of adult mice.

## 1 Introduction

Inner ear disorders, deafness, and balance disequilibrium are among the most common disabling conditions; indeed, it could be argued that the inner ear is responsible for a greater incidence of disability than any other organ system in the body. Such disorders become increasingly prevalent with age. More than 40% of those >50 years old have some degree of clinically significant hearing loss and this percentage increases dramatically to 70% in those >70 years of age (http://www.actiononhearingloss.org.uk/your-hearing/about-deafness-and-hearing-loss/statistics.aspx). Dizziness is the most common reason for visits to a general practitioner among those >60 years old, and balance disequilibrium is a significant contributor to falls in the elderly (Davis, 2009; Department of Health, UK, 1999; Gates et al., 2008; Herdman et al., 2000; Jarvinen et al., 2008; Macias et al., 2005; Pothula et al., 2004). The most common cause of hearing impairment and balance dysfunction is the loss of the mechanosensory “hair” cells from the sensory epithelia of the cochlea, the hearing organ (Brown et al., 1989; Hawkins, 1973; Kujawa and Liberman, 2006; Ohlemiller, 2004; Prosen et al., 1981), and the vestibular system, which subserves balance (Baloh et al., 2001; Rauch et al., 2001; Wright, 1983). In nonmammalian vertebrates, birds, reptiles, amphibians, and fish, lost hair cells are replaced spontaneously by new ones (Adler and Raphael, 1996; Corwin and Cotanche, 1988; Cotanche, 1987; Ryals and Rubel, 1988; Stone and Cotanche, 2007; Taylor and Forge, 2005). These arise from the nonsensory supporting cells that surround each hair cell. There is no regeneration of hair cells in the mammalian cochlea, so auditory deficits are permanent. Regeneration of hair cells in the vestibular system of mammals has been reported (Forge et al., 1993, 1998; Kawamoto et al., 2009; Warchol et al., 1993), but the capacity to do so spontaneously is limited severely, so that vestibular functional deficits resulting from hair cell loss are also permanent. It is also not known whether the capacity to regenerate hair cells is retained in humans, or whether it declines with age.

Studies of the inner ears of animals have revealed pathologic processes that lead to hair cell death (Baker et al., 2014; Cheng et al., 2005; Esterberg et al., 2013; Forge and Li, 2000; Schacht et al., 2012). From such understanding, possibilities for therapeutic interventions to protect hair cells from lethal damage are being investigated. The potential for replacing lost hair cells with new ones, either through inducing endogenous regenerative mechanisms similar to those that occur spontaneously in nonmammalian vertebrates, or via exogenous processes such as application of precursors derived from stem cells, has also been suggested. Although it is assumed generally that cellular and molecular mechanisms observed in the inner ear tissues from animals are applicable to human tissue, both scientifically and for translational purposes, this requires validation. In humans, the inner ear is encased within the temporal bone, reputed to be the hardest bone in the body, at the base of the skull. The consequent inaccessibility of human inner ear tissue limits severely possibilities for their direct experimental manipulation.

There are some occasions, however, when viable inner ear tissue from humans becomes available for experimentation. During surgery for excision of vestibular schwannomas (also known as acoustic neuromas), the vestibular portion of the inner ear is exposed, removed, and usually discarded, but it can be harvested for study. Mature vestibular sensory tissues from several different animal species have been successfully maintained ex corporeally in organotypic culture for ≥4 weeks, thereby enabling direct experimental studies of the tissue (Cunningham, 2006; Li and Forge, 1995), and studies of human inner ear tissue maintained in culture have been reported (Kesser et al., 2008; Warchol et al., 1993). However, few studies have performed long-term cultures, and previous studies have been limited to a small number of samples.

For this study, we established a consortium of surgeons who work at the major centers in England where trans-labyrinthine surgery to remove acoustic neuromas is performed to obtain a consistent supply of relatively large numbers of samples. Ultimately, our aim is to use human vestibular tissue in experimental studies for hair cell protection and regeneration as potential therapies to ameliorate age-related functional deficits. In this study, we assess characteristics of the human vestibular sensory tissue and its maintenance in culture. We report on hair cell pathologies that suggest effects of ageing and the novel observation of the presence of immature hair bundles on human utricular epithelia from elderly people. This work establishes the explanted human utricular macula from trans-labyrinthine surgery as a model in vitro system for translational inner ear studies.

## 2 Methods

### 2.1 Harvesting and collecting tissue

Samples were obtained from patients undergoing excision of a vestibular schwannoma (acoustic neuroma) via a trans-labyrinthine approach. Tissue was collected anonymously after obtaining informed consent from the patient, and with the approval of the UK National Health Services (NHS) and University College London (UCL) Ethics committees and the local NHS research and development administrations at the National Hospital for Neurology and Neurosurgery, UCLH NHS Trust, London; Addenbrooke's Hospital, Cambridge; Queen Elizabeth Hospital Birmingham; Manchester Royal Infirmary; and Salford Royal Infirmary. Patient gender and age were recorded. A standardized procedure for accessing and removing the vestibular sensory tissue that could be used by all the surgeons who were involved in the harvesting was developed. Patients ranged between 16 and 81 years old (mean, 50).

Upon removal, tissue was transferred immediately to the medium to be used for long-term maintenance and brought to the laboratory. Samples from London, Cambridge, and Birmingham were collected personally and transported to the laboratory arriving within 20 minutes, approximately 2 and 3.5 hours, respectively, after removal from the patient. Samples from Manchester and Salford were sent by mail (using the Royal Mail Safebox containers), usually arriving within 2 days after the surgery, but sometimes later.

### 2.2 Maintenance and manipulation of vestibular tissue in explant culture

To culture vestibular tissue after transportation, utricles and cristae were incubated in 0.5 mL of Minimal Essential Medium (MEM) glutamax (Gibco) with 1% *N*-2-hydroxyethylpiperazine-*N*'-2-ethanesulfonic acid (HEPES) and 10% fetal bovine serum (Hyclone) free-floating in a 24-well plate for ≤4 weeks at 37°C, 5% CO_2_. Ciprofloxacin and amphotericin B were added to the medium to prevent microbial contamination. Medium was changed (50%) on alternate days. To explore the responses of supporting cells to hair cell loss, hair cells were ablated by exposure to 2 mmol/L gentamicin for 48 hours.

### 2.3 Assessment and processing of tissue

On arrival in the laboratory, tissue was examined for suitability of use. Tissue was transferred to a 35-mm petri dish with fresh medium and examined under a dissecting microscope. Our criteria for use included a clearly defined utricle with intact epithelium on the upper surface. Fanned-out nerve tracts on the underside assisted identification. Undamaged cristae within ampullae were also retained.

To assess the viability of the isolated human vestibular tissue, some samples at random were rinsed in HEPES-buffered Hanks buffered salt solution (HBHBSS) pH 7.3 exposed for not 30 seconds to 20 μmol/L FM1-43 or FM1-43FX (Molecular Probes, Invitrogen, Paisley), a fixable derivative of FM1-43, in serum-free medium, MEM with glutamax, and 1% HEPES. Samples were then washed twice in HBHBSS before examination on an inverted microscope (Nikon) and images acquired using a Hamamatsu Orca camera with Simple PCI software. Brief exposure to FM1-43 labels viable hair cells, but nonsensory cells in the tissue including supporting cells remain unlabeled (Gale et al., 2001).

Tissue was fixed for immunohistochemistry or fluorescent labeling in 4% paraformaldehyde (PFA) in phosphate-buffered saline (PBS) for 90 minutes. Tissue was then prepared for examination as whole mounts or as cryosections. For whole mounts, after fixation utricles and cristae were rinsed in PBS, permeabilized in 0.5%Triton in PBS for 20 minutes, and placed in blocking solution (10% goat serum in PBS with 0.15% triton X-100). Tissue was incubated in primary antibody overnight at 4°C rinsed thoroughly in several changes of PBS and then incubated with appropriate secondary antibody conjugated to a fluorophore for 90 minutes at room temperature. Samples were mounted onto slides using Vectashield with DAPI (Vector Laboratories) to label nuclei and examined and imaged on either a Zeiss Axiovert or Zeiss LSM 510 confocal microscope. For cryosections, fixed tissue was incubated overnight at 4°C in 30% sucrose solution and embedded in low-temperature-setting agarose, oriented and mounted on specimen support stubs using optical coherence tomography. Sections were cut at 15 μm and collected on polylysine coated slides (Polysine, VWR). Labeling was performed as for whole mounts.

Hair cell markers were as follows: antibodies to calretinin (1:50, Invitrogen, Paisley); myosin VI (1:200, Sigma, Poole); and myosin VIIa (1:100, Developmental Hybridoma Bank, Iowa). Supporting cells were examined for the expression of proteins using the following antibodies: a rabbit polyclonal against sox-9 (1:100, Millipore); a guinea pig polyclonal against l-glutamate/l-aspartate transporter (GLAST; 1:75, Millipore); a polyclonal against connexin (CX)30 (1:200 Zymed) and a rabbit polyclonal against CX26 (Gap 28H, kind gift Prof WH Evans; these connexin antibodies having previously been tested for specificity in a HeLa cell homologous expression system [Kelly et al., 2011]); a rabbit polyclonal against the c-terminus of human CX43 (Sigma); a rabbit polyclonal against Kir 4.1 (1:100, Alomone Labs, Jerusalem); and a goat polyclonal (1:200, Abcam, Cambridge, UK) and a rabbit polyclonal (1:200, kind gift from Thomas Jentsch, Berlin) against KCC4. A fluorescent phalloidin conjugate was added at 1 μg/mL to the secondary antibody solution to label filamentous actin.

Harvested tissue was also prepared for scanning electron microscopy (SEM) and for thin sections for transmission electron microscopy (TEM). Tissue was fixed in 2.5% glutaraldehyde in 0.1 mol/L cacodylate buffer for 2 hours and post-fixed in 1% OsO4 in cacodylate buffer for 1.5 hours. For SEM, the samples were then processed by the repeated thiocarbohydrazide-osmium procedure (Davies and Forge, 1987) before dehydration in an ethanol series and critical point drying. Samples were mounted on support stubs using silver paint and sputter-coated with platinum before examination. Digital images were collected. For thin sectioning, tissue was partially dehydrated to 70% ethanol and “en bloc”-stained in a saturated solution of uranyl acetate in 70% ethanol before further dehydration and embedding in plastic. Sets of serial thin sections were cut at several depths through the tissue. Some sections were mounted on formvar-coated single-slot grids to enable uninterrupted views across the section. The sections were stained with aqueous uranyl acetate and lead citrate and examined in a JEOL 1200EXII instrument operating at 80 kV. Digital images were collected with a Gatan camera.

Some specimens processed and examined by SEM were subsequently carefully removed from support stubs and embedded in plastic for thin sectioning for TEM. The tissue samples were separated from the SEM support stub by immersion in methyl isobutyl ketone (silver paint diluent) to dissolve the silver paint adhesive and washed in acetone before embedding in plastic by routine procedures. Some of these thin sections were mounted on silicon wafers (Agar Scientific) and examined in the SEM using back scatter detection for imaging. The silicon wafers were mounted on specimen supports using silver paint as an adhesive and conductive film. This procedure enabled examination of the entire tissue section without the obstruction of segments by grid bars as occurs with TEM.

### 2.4 Dye transfer in vibratome slices

Utricular maculae were enrobed in low gelling temperature agarose (Sigma) and slices 200 μm thick were cut on a vibratome using procedures described elsewhere (Forge et al., 2013; Jagger and Forge, 2006; Jagger et al., 2014). The slices were maintained in artificial perilymph (150 mmol/L NaCl, 4 mmol/L KCl, 2 mmol/L MgCl2, 1.3 mmol/L CaCl2, 10 mmol/L HEPES, and 5 mmol/L glucose, pH adjusted to 7.3 with NaOH). Supporting cells were located within the slice and an individual cell identified for patch clamping. Neurobiotin and fluorescein-conjugated dextran were injected together into the cell via the patch clamp electrode whilst performing whole cell patch clamp recording. The slice was subsequently fixed for 30 minutes in 4% PFA and processed for neurobiotin detection. Briefly, tissue was permeabilized (0.1% Triton X-100 for 40 minutes), and blocked (0.1 mol/L l-lysine, at 35°C for 40 minutes), before incubation for 2 hours in Alexa Fluor 555-conjugated streptavidin (1:1000; Invitrogen, Carlsbad, CA).

### 2.5 Mouse tissues

Some comparisons were made with utricular maculae of mice. Tissue of early postnatal (P5-P7) and young adult (30–40 days old) animals was obtained either from C57Bl/6 or CBA/Ca mice. Utricular maculae from aged animals were obtained from CBA/Ca mice, 22–27 months old. The tympanic bullae of the animals were isolated and opened. For studies of the viable tissue, utricular maculae were isolated under sterile conditions and transferred to maintenance medium as described. For fixation, the round and oval windows of the cochlea were opened and the vestibule widely exposed, and a small piece of bone removed from the cochlear apex. Fixative, either 4% PFA in PBS or 2.5% glutaraldehyde in 0.1 mol/L cacodylate buffer, was gently injected into the inner ear via these openings. The opened bullae were then immersed in fixative for 2 hours at room temperature. The samples were decalcified in 4% EDTA in the appropriate buffer before dissection of the inner ear and isolation of the utricular maculae. Processing for immunohistochemistry, SEM, or thin sectioning was as described. Some additional mouse samples were rapidly freeze-fixed without prior chemical treatment by high-pressure freezing, and subsequently processed by freeze substitution using procedures outlined elsewhere (Bullen et al., 2014).

## 3 Results

### 3.1 Viability of harvested human vestibular tissue

**Fig. 1:**
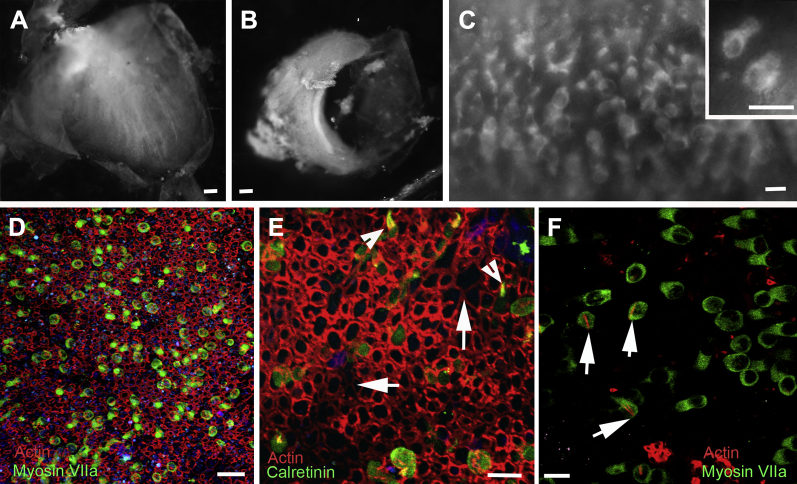
(A–C) FM1-43 uptake. (A) Utricular macula. (B) Crista. Labeling across the entire tissue surface. (C) Utricular macula. Dye is confined to cells with the shapes and pattern of distribution of hair cells. Inset shows cells that take up the dye have the shape expected of hair cells. (D) Hair cells labeled for myosin VIIa (green) are distributed across the epithelium (filamentous actin labeled with phalloidin-TRITC [red]). (E) Hair bundles (arrowheads) and hair cell bodies labeled for calretinin. Phalloidin labeling of filamentous actin (red) in hair bundles (arrowheads) and in wide bands at the junctions between supporting cells, but in some regions from which hair cells have been lost and the lesions closed by expansion supporting cells, the junctional actin bands are thinner (arrows). (F) Phalloidin labeling reveals actin “rods” through the bodies of some hair cells (arrows) in fresh-fixed tissue. Scale bars: (A, B) 20 μm; (C) 10 μm; inset (10) μm; (D) 20 μm; (E, F) 10 μm.

Entire, intact utricles (Fig. 1A) and cristae (Fig. 1B) could be isolated. Most samples of the human utricular maculae arrived without the covering otoconial membrane. This membrane seemed to have been lost during harvesting. The viability of tissue samples was assessed upon arrival in the laboratory by determining uptake of FM1-43. This dye is taken up through the transduction channels and possibly via endocytosis, providing a marker for viable hair cells (Gale et al., 2001). In utricular maculae and cristae exposed briefly to FM1-43 (Fig. 1A, B), cells throughout the entire epithelium were fluorescently labeled, and higher power (Fig. 1C) revealed the labeling was confined to cells with a morphology consistent with that of hair cells. All 5 samples examined by this method showed labeled hair cells distributed across the entire epithelium, including 1 sample that had arrived 4 days after removal from the patient.

### 3.2 Hair cell characteristics

A number of characterized hair cell markers—myosin VI, myosin VIIa, and calretinin—were used to identify hair cells in fixed tissue. Immunolabeling of utricles with antibody to myosin VIIa revealed expression of the protein in the cytoplasm and cuticular plate of the hair cell (Fig. 1D). Antibody to the calcium binding protein, calretinin, labeled both the cytoplasm and hair bundles and identified the presence of hair bundles (Fig. 1E). However, throughout the tissue there were a number of cells labeling positively for myosin VIIa, but in which hair bundles were not labeled (data not shown). Phalloidin labeling of filamentous actin also revealed in some hair cells actin-rich cables running diagonally the length of the hair cell body (Fig. 1F). These resembled cytocauds, a phenomenon previously described in type I vestibular hair cells of the waltzing guinea pig and mutant mice (Kanzaki et al., 2002; Sobin et al., 1982). In human utricular epithelia, these actin cables were seen to extend the length of the hair cell but did not attach to the basement membrane. Cytocaud-like labeling in hair cells was found more consistently in remaining hair cells after exposure of the tissue to gentamicin (see below).

**Supplementary Fig. 1:**
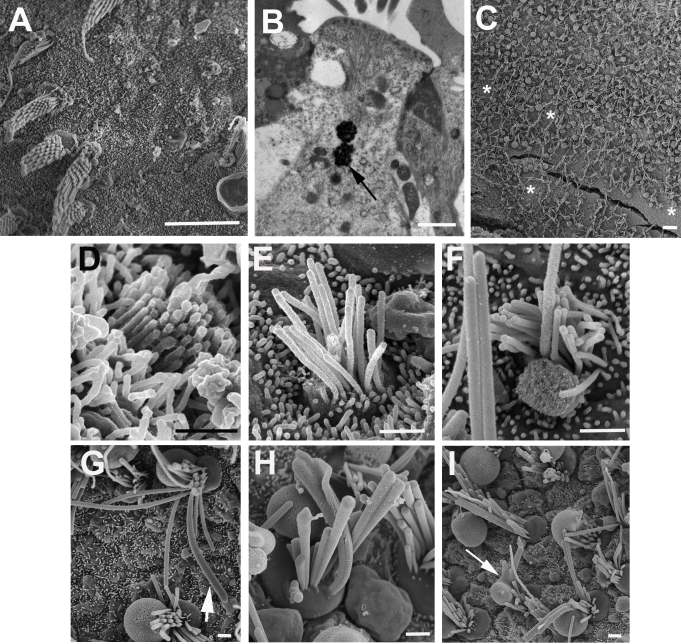
Mouse utricular maculae. (A) A 22-month-old CBA/Ca mouse. Stereocilia of hair bundles in orderly arrays. Hair cells missing over a large area where supporting cells have closed the lesions. (B) A 24-month-old CBA/Ca mouse. Longitudinal section of hair cell with granules (arrow) of an appearance consistent with lipofuscin. (C) A 24-month-old CBA/Ca mouse. The macula is covered with hair cells, but there are many regions where hair cells are missing (asterisks) throughout the tissue. (D–F) Immature bundles. (D) Immature hair bundle in early postnatal (P4) C57Bl/6 mouse. Stereocilia are of equal height and cross-linked. (E, F) Immature-appearing hair bundles in old mice: 24-month-old CBA/Ca. (G–I) Stereocilia anomalies in old (24 months) CBA/Ca mice. (G) Giant, fused stereocilia (arrow). (H) Multiple fusions and swellings along shaft and at tip. (I) Fusion and enlarged swelling (arrow). Scale bars: (A) 10 μm; (B) 1 μm; (C) 10 μm; (D–I) 1 μm.

**Fig. 2:**
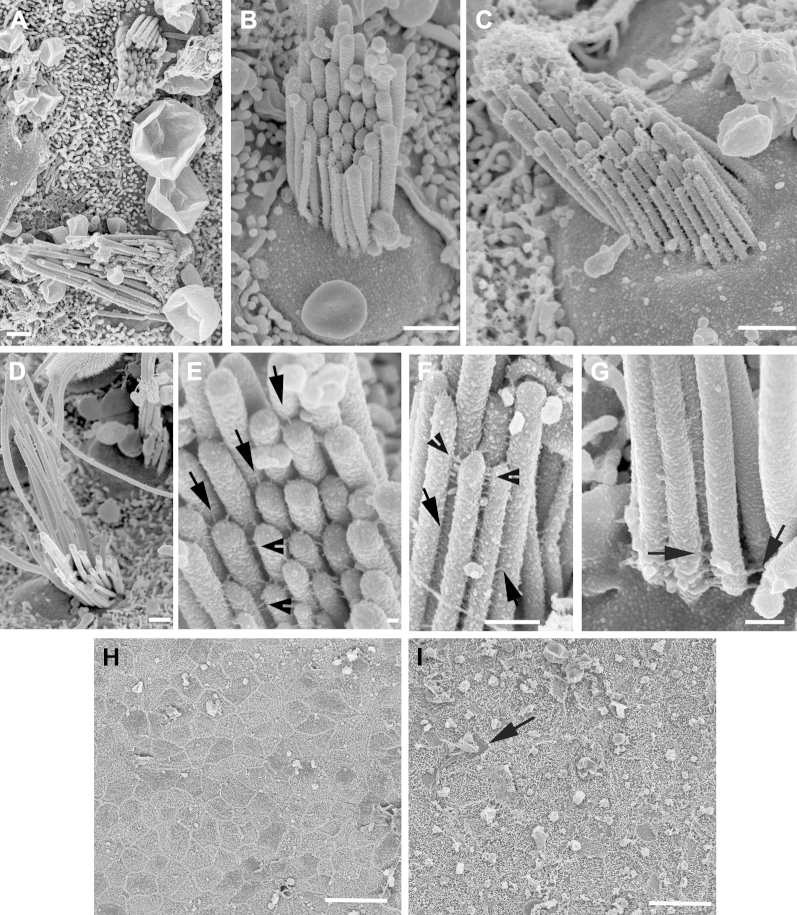
(A–G) Hair bundles in recently excised utricular maculae. (A) Adjacent bundles of differing height. (B–D) Different bundle “types.” In (B) the stereocilia, especially the shorter ones are generally thicker than those in (C), but there is variability in the width of stereocilia of similar height within individual bundles. In (D), the tallest stereocilia are markedly longer than the shorter ones. Although there is height gradation across the bundles, there is variability in the differential lengths of adjacent stereocilia. (E–G) Stereocilia cross-links. (E) Tip-links (arrows) and top connectors (arrowheads). (F) Top connectors (arrowheads) and shaft connectors (arrows). (G) Ankle links (arrows). (H, I) Almost complete loss of hair cells in utricular maculae from elderly people. (H) An 80-year-old woman; the surface consists almost entirely of a pavement of supporting cell surfaces which are covered with short microvilli. (I) A 65-year-old man. A single hair bundle (arrow) is evident across the field. Scale bars: (A–D) 1 μm; (E) 0.1 μm; (F) 0.5 μm; (G) 0.25 μm; (H, I) 10 μm.

SEM revealed morphologic variability among hair bundles and preservation of expected structural details (Jeffries et al., 1986). There was a clear variation between hair bundles in the lengths of the longest stereocilia, even between those on closely adjacent hair cells (Fig. 2A). In individual hair bundles, there was also an apparent variation in the widths of the stereocilia (Fig. 2B, C), but because most stereocilia showed the expected tapering at their basal end where they arise from the apical surface of the cell, it seems unlikely that the width variation is a consequence of fusion (fused stereocilia are usually irregular in shape). Most markedly, in nearly all individual hair bundles, although stereocilia generally graduated in height, the regularity of the height differential that is commonly seen in hair bundles elsewhere, including those in the utricle of aged mice (Supplementary Fig. 1A), was much less evident; there were often clear differences in the height (and sometimes the width), of adjacent stereocilia that seemed to be in the same “row,” although the definition of stereociliary rows was not always clear cut (Fig. 2B–D). Nevertheless, all the various types of links between stereocilia in an individual bundle that have been described (Nayak et al., 2007; Richardson et al., 2011) were identifiable: tip links, which extended from the tips of some shorter stereocilia to adjacent longer stereocilia approximately in the direction of the bundle's polarity (Fig. 2E); top connectors (Fig. 2E, F) extending laterally between stereocilia at their distal ends; shaft connectors, which seemed thinner than the top connectors (Fig. 2F); and ankle links in the region of the taper at the proximal end of the stereocilia (Fig. 2G). The apical tip of some stereocilia to which a tip link was attached was round on some stereocilia, whereas others had a more pointed, “tented” appearance.

**Fig. 3:**
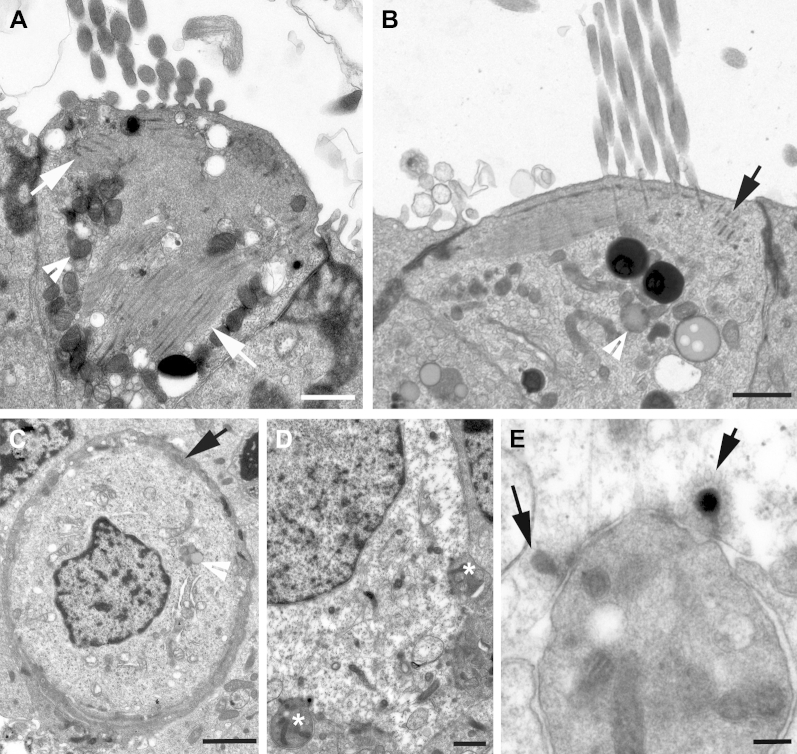
Thin sections of hair cells in utricular macula. (A, B) Striated bodies (arrows) associated with cuticular plate: type 1 (A) and type 2 (B) hair cell. (C, D) Innervation. (C) Thin afferent calyx (arrow) surrounding body of type 1 hair cell. (D) Base of type 2 hair cell showing 2 bouton nerve endings (asterisks). (E) Two synaptic ribbons (arrows) opposite a single afferent nerve bouton. Arrowheads in (A–C) indicate lipofuscin granules. Scale bars: (A–D) 1 μm; (E) 0.2 μm.

Thin sections showed prominent striated bodies just below and around the cuticular plate (Friedmann et al., 1965; Sans, 1989; Vranceanu et al., 2012) to be present in types 1 and 2 hair cells (Fig. 3A, B), but they were not apparent in all hair cells; in many instances, even after examination of several sections of the same hair cell in a semiserial section series striated bodies were not revealed. Neuronal elements, especially the calcyeal endings around the bodies of type 1 hair cells, were often degenerating, suggesting a particular vulnerability of these neurones in excised tissue, but when identifiable the calcyeal endings were relatively thin (Fig. 3C) and mitochondria were often prominent (Fig. 7D). Synaptic ribbons were present in hair cells, and in type 2 hair cells often >1 ribbon opposed an afferent bouton ending (Fig. 3D, E). It has been reported that hair cells possessing >1 synaptic ribbon apposing a single afferent nerve ending are more prevalent across the striolar region of the utricle (an inner strip of the macula epithelium where hair bundle orientation changes by 180°) rather than at the periphery (Lysakowski and Goldberg, 1997); however, although the hair cells with multiple ribbons were present in the more central regions of the epithelium, we were unable to identify the striolar region definitively in our thin-sectioned samples.

In all samples examined by thin sectioning, hair cells and supporting cells both commonly showed numerous electron dense inclusions reminiscent of lipofuschin granules (Fig. 3B–E; Gleeson and Felix, 1987). Similar structures were observed in hair cells and supporting cells of elderly CBA/Ca mice (Supplementary Fig. 1B).

In some samples from the oldest patients (70–80 years old), SEM revealed extensive loss of hair cells. Across the entire macula the tissue surface seemed primarily as a pavement of cells with short microvilli characteristic of the apical surfaces of supporting cells (Fig. 2H), with only an occasional hair cell evident (Fig. 2I). The loss of hair cells was much more extensive than that observed in the utricular maculae of the oldest CBA/Ca mice examined (27 months old) where, although there was a clear reduction in the density of hair bundles compared with young animals, many hair cells were still present but with areas devoid of hair cells and filled with the heads of supporting cells scattered across the maculae (Supplementary Fig. 1A, C). A preferential loss of hair cells from the striolar region, as occurs for example after exposure to aminoglycosides, was not obvious in the utricular maculae of the aged mice.

### 3.3 Immature hair bundles are present in the adult human utricular maculae

**Fig. 4:**
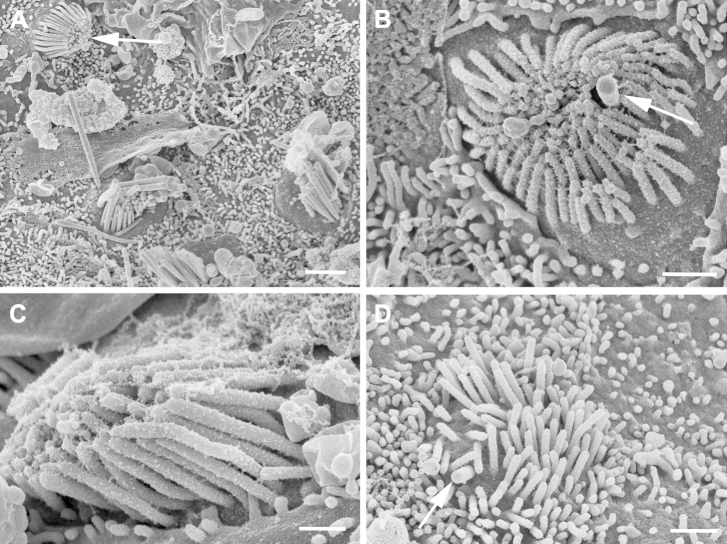
Bundles with immature characteristics in utricular maculae. (A) Bundle with short stereocilia of equal height (arrow) among mature-appearing bundles in tissue from a 60-year-old woman. (B) Stereocilia of approximately equal height cover almost the entire apical surface of the cell, are extensively cross-linked and surround the thicker kinocilium (arrow); from a 65-year-old man. (C) Cluster of short stereocilia occupying entire cell surface are extensively cross-linked; from a 65-year-old man. (D) Cluster of elongated “microvilli” with eccentrically positioned thicker projection, reminiscent of a kinocilium (arrow); from 85-year-old woman. Scale bars: (A) 5 μm; (B–D) 1 μm.

In ≥3 utricular epithelia, among mature bundles there were also hair bundles with short stereocilia (Fig. 4A–D) that resembled the immature bundles seen to arise during regeneration of hair cells in guinea pig and mouse vestibular epithelia after gentamicin-induced hair cell loss (Forge et al., 1993, 1998; Kawamoto et al., 2009), and those in the utricular maculae of embryonic and early postnatal mice (Denman-Johnson and Forge, 1999; Supplementary Fig. 1D). The stereocilia of these bundles were of approximately equal length, between 1 and 2 μm, and lacked the stepwise graduation evident in more mature bundles (Fig. 4B, C). As is common in immature hair bundles, the stereocilia were extensively cross-linked along their length. A clearly defined, thicker kinocilium was located off center to 1 side of the bundle, often surrounded by stereocilia. The bundles occupied almost the entire apical surface of the cell, which was characteristically smaller than that of cells bearing mature bundles where the bundle occupied only a portion of the apical surface. Bundles with these immature characteristics were observed in tissue from elderly (>60 years old) patients, and most remarkably, such a bundle was apparent in a region where almost no other hair bundles were present, on the utricular macula harvested and directly fixed for SEM examination from a patient >80 years old (Fig. 4D). Stereociliary bundles of similar immature characteristics were also observed in the utricular maculae of aged (>22 months old) CBA/Ca mice (Supplementary Fig. 1E, F). This development suggests that even vestibular epithelia of aged patients retain a capacity to generate new bundles.

### 3.4 Hair cell pathologies

**Fig. 5:**
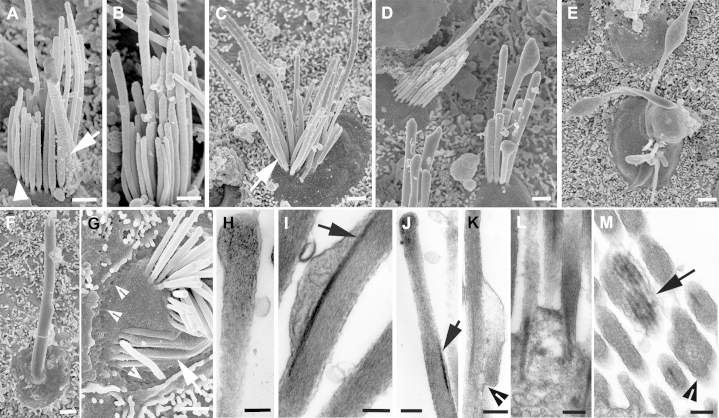
Stereociliary pathologies. (A–G) Scanning electron microscopy. (A) Stereocilia of varying width along length (arrowhead) and kinocilium fused with stereocilium (arrow). (B) Individual stereocilia vary in width along their length. (C) Stereocilia show swellings along the shaft (arrow). (D) Fused stereocilia and various swellings at distal ends. (E) Fused, elongated, and swollen stereocilia. (F) Single fused “giant” “stereocilium” on an individual hair cell. (G) Stereocilia apparently internalized at the apical surface of the hair cell (arrow), which shows numerous vesicle openings (arrowheads) around the periphery of the of the luminal surface. (H–M) Thin sections. (H) Swollen apical tip of a stereocilium. (I) Swelling along the shaft of the stereocilium in which cytoplasmic-like material is enclosed by the stereociliary membrane and a dense rod-like structure (arrowed) is located at the periphery of the stereociliary filament bundle. (J) Stereocilium showing a thinning along its shaft and a dense rod structure within the shaft. (K) Stereociliary fusion of 1 stereocilium at the site of a swelling along the shaft of another (arrowhead). (L) Hair cell apical membrane in the region between the points of insertion of stereocilium is detached from the cuticular plate and is rising between and fusing with adjacent stereocilia. (M) Fused stereocilia (arrowhead) and fusion between stereocilia and kinocilium (arrow). There seem to be 2 ciliary axonemes within the limiting membrane of the kinocilium. Scale bars: (A–G) 1 μm; (H, I) 0.2 μm; (J, K) 0.5 μm; (L, M) 0.2 μm.

Where hair bundles were preserved in samples fixed immediately upon arrival in the laboratory and prepared for electron microscopy (5 SEM; 4 TEM), a variety of stereociliary anomalies were apparent. Individual stereocilia had abnormal shapes, varying in width along their length and often tapered toward their apical ends (Fig. 5A, B). Other stereocilia showed swellings, sometimes quite large, at some point along the shaft, particularly at their distal ends (Fig. 5C–E) to create bizarre forms. Stereocilia were often fused and/or elongated, sometimes into single, large, thick projections from the apical surface (Fig. 5F). The apparent internalization of stereocilia was also evident; the apical plasma membrane covered impressions of stereocilia at the apical surface (Fig. 5G). Thin sections showed swellings in stereocilia were not composed of actin filaments, but seemed to be filled with cytoplasmic-like material enclosed between the limiting membrane and the actin filament bundle (Fig. 5H, I). Sectioning also revealed dense filamentous structures located within the stereociliary shaft (Fig. 5I, J) reminiscent of the densities usually present at the proximal ends of stereocilia, where they taper and that run from the stereocilia into the cuticular plate. Thinning of stereocilia along the shaft (Fig. 5J) and fusion of stereocilia (Fig. 5K, M) were also apparent. Where fusion occurred, cytoplasmic-like material sometimes seemed to become incorporated within the region of fusion (Fig. 5K). The region of apical membrane between the tapering ends of stereocilia was sometimes detached from the cuticular plate and located above the point of the taper, suggesting initial stages of stereociliary fusion. Fusion of the kinocilium with stereocilia was also evident (Fig. 5A, M) and there sometimes seemed to be >1 microtubular shaft enclosed within the limiting membrane of the kinocilium (Fig. 5M); whether this was the result of 2 shafts within a single kinocilium, or from fusion of a single kinocilium bent back on itself was not clear, but the latter condition was not observed in SEM samples. Thin sections revealed that hair cells displaying stereociliary peculiarities were otherwise normal, with no obvious indications of degeneration, suggesting the pathology may be affecting stereocilia specifically.

Stereociliary anomalies similar to those observed by SEM in the human tissue were also evident in utricular maculae of aged CBA/Ca mice (Supplementary Fig. 1G–I), but not in those of neonatal or young adults prepared under identical conditions. This indicates that the stereociliary anomalies are not a consequence of tissue preparation, but do suggest that they are age related.

### 3.5 Survival of hair cells in vitro

**Fig. 6:**
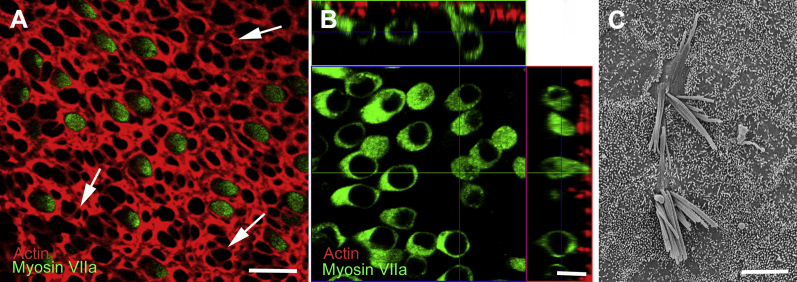
Utricular maculae maintained in organotypic culture. (A) Seven days in vitro. Confocal projection image of apical surface of whole mount preparation. Hair cells, labeled for myosin VIIa (green), are distributed throughout the epithelium. Phalloidin labeling of filamentous actin delineates the junctional regions around the necks of supporting cells. Where some hair cells have been lost, the heads of supporting cells have expanded to close the lesions producing characteristic scar formations where the junctional actin bands are thinner than elsewhere (arrows). (B) Nine days in vitro. Confocal image of single optical section with orthogonal projections. Both flask-shaped type 1 and more cylindrical type 2 hair cells are retained. (C) Twenty-one days in vitro. Scanning electron microscopy shows hair bundles with long stereocilia, but some evidence of fusion after this period of ex corporeal maintenance. Scale bars: (A) 10 μm; (B, C) 5 μm.

Explanted utricles were maintained in medium for periods of ≤28 days. Antibodies to myosin VIIa demonstrated the survival of the hair cells within the sensory epithelium for long periods of time (Fig. 6A, B). DAPI labeling of nuclei showed few pyknotic nuclei among the hair cell population and none at the level of supporting cell nuclei, confirming survival of these cells during ex vivo maintenance. Hair cells with flask-like morphology, that is, those with long necks, extended to the luminal surface of the epithelium. Other myosin VIIa–positive cells with a more cylindrical cell body were also apparent (Fig. 6B). SEM revealed that hair bundles could be maintained for ≤28 days. Although stereocilia in an individual bundle were sometimes fused, they were often long and with recognizable persistence of gradation in height (Fig. 6C). Similar stereociliary fusion is found commonly in explant cultures of utricular maculae from mice and other animals maintained for prolonged periods ex vivo (Li and Forge, 1995). Supporting cell surfaces were covered in short microvilli, because they were in tissue fixed immediately upon arrival in the laboratory.

### 3.6 Hair cells can survive without hair bundles and do not regrow them

**Fig. 7:**
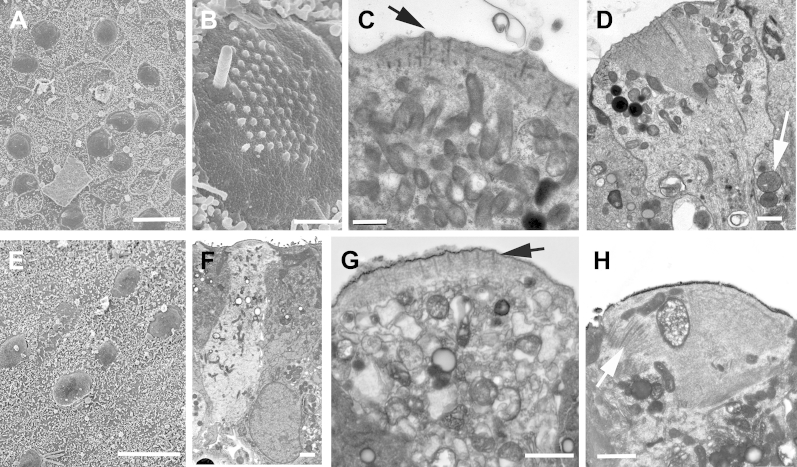
Hair cells survive without hair bundles. (A–D) Utricular macula fixed immediately after harvesting. (A) Hair cells devoid of bundles, many with impressions of the insertion points of stereocilia at the apical surface, are scattered across the epithelium. (B) Scanning electron microscopy (SEM) of the surface of an individual hair cell. Short stumps of the stereocilia and of the kinocilium are covered over; their internal structure is not exposed. (C) Thin section of the apical end of a type 2 hair cell shows continuous plasma membrane mounded over the stereociliary rootlets descending into the cuticular plate (arrow). The cytoplasm below the cuticular plate shows no evidence of degenerative changes. (D) In a type 1 hair cell, the apical plasma membrane is continuous over the cuticular plate with no indication of disruption at the sites of the stereociliary rootlets. There is no indication of cellular degeneration in the cytoplasm below the cuticular plate. The arrow points to large mitochondria in the afferent nerve calyx. (E–H) Four days in vitro. (F–H) Images collected by backscatter detection in the SEM of sections of the same sample examined by conventional SEM shown in panel. Hair cells without bundles persist in the epithelium (E). The hair cells show no obvious evidence of degeneration (F). At the apical ends of the cells ([G], type 2 hair cell; [H], type 1), the cuticular plate with persisting stereociliary rootlets (arrow in [G]) is covered by a continuous plasma membrane, and striated bodies are retained (arrow [H]). Scale bars: (A) 10 μm; (B) 1 μm; (C) 0.5 μm; (D) 1 μm; (E) 10 μm; (F–H) 1 μm.

In most samples, ≥2 of which showed FM1-43 uptake into hair cells, SEM revealed that many hair cells were devoid of hair bundles (Fig. 7A; as previously reported [Ylikoski, 1980]), but retained remnants of the stereocilia and kinocilia as short stubs at the apical surface (Fig. 7B). Unlike SEM images of the apical surface of hair cells in which stereocilia have been broken at their distal ends during preparation when the internal structure at the junction of the stereocilium with the apical membrane of the cell is exposed, these stereociliary remnants were usually closed with what seemed to be a continuity of the apical plasma membrane. Other cells with an apical surface area and in a location consistent with a hair cell showed completely smooth surfaces. Thin sections showed intact hair cells without apparent stereocilia, but with an intact cuticular plate covered by a continuous, uninterrupted apical plasma membrane mounded over the sites of stereociliary rootlets (Fig. 7C). Other intact hair cells had a completely smooth, continuous membrane covering the cuticular plate in which stereociliary rootlets were present (Fig. 7D). These observations suggest that, with loss of stereocilia, the lesion in the apical membrane can be closed efficiently and sealed by membrane sufficiently rapidly to prevent cell death.

In some samples maintained in explant culture for ≤28 days, SEM showed cells with smooth surfaces, with a surface area size and in a distribution, consistent with that of hair cells (Fig. 7E). After conventional SEM examination, these samples were embedded in plastic. Serial thin sections were cut, mounted on silicon wafers, and examined by back scatter detection in the SEM. This procedure enabled examination of the entire width of the utricle and assessment of most of the hair cells contained within it. It revealed that all the hair cells were intact in the absence of the hair bundle (Fig. 7F–H), with no evidence of significant degeneration and that there were no smooth surfaced apical remnants of hair cells, that is, every smooth surface observed by SEM denoted a hair cell that was intact other than the absence of a hair bundle. There was no evidence of immature hair bundles arising on cells with a surface area of mature hair cells. In samples maintained in normal medium for ≤4 weeks, only hair cells with recognizable hair bundles, sometimes with the stereocilia fused as described (Fig. 6C), or cells with a smooth apical surface, were apparent. This may suggest that hair cells not only can survive without bundles, but also that at least over the time period examined and under conditions of culture, mature, intact hair cells do not regenerate spontaneously their lost bundles.

### 3.7 Gentamicin-induced hair cell loss

**Fig. 8:**
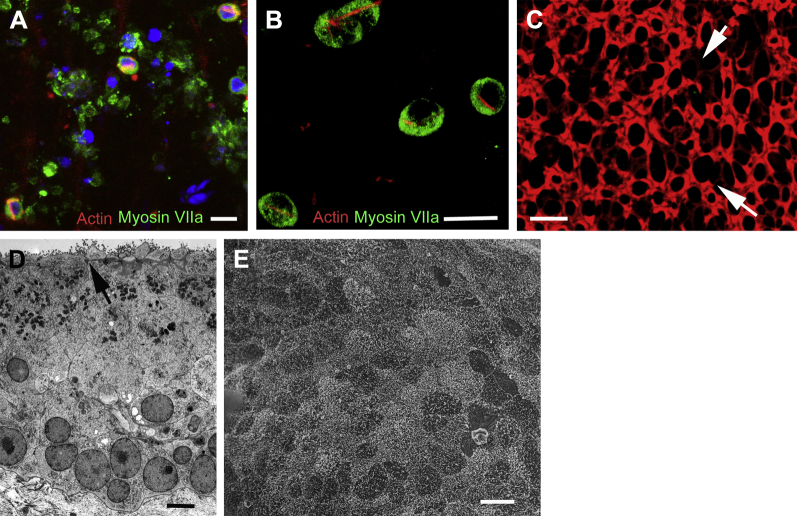
Utricular maculae after incubation with 2 mmol/L gentamicin for 48 hours. (A, B) Two days after the end of gentamycin exposure. (A) Few intact hair cells remain, but myosin VIIa–labeled debris and some pyknotic nuclei (DAPI, blue) are present at the level of hair cell nuclei. (B) Most remaining, apparently intact hair cells (positive labeling for myosin VIIa) contain an actin cable running through the length of the cell. (C) Seven days after gentamicin exposure. Phalloidin labeling of f-actin at the cell junctions, shows numerous “scar” formations in the positions once occupied by hair cells where supporting cells have closed the lesions repair in which the junctional actin bands are relatively thin. (D) Four days after gentamicin exposure. Thin section shows no hair cells are present and supporting cells, with nuclei predominantly positioned at the basal aspect of the epithelium, have repaired efficiently the epithelium. The arrow indicates a relatively simple adherens junctional assembly. (E) Twenty-one days after gentamicin exposure. Scanning electron microscopy reveals that the apical surface of the epithelium is composed of a pavement of supporting cell apices, with no indication of the presence of remaining hair cells or of immature hair bundles. Scale bars: (A–C) 10 μm; (D) 5 μm; (E) 10 μm.

Incubation of the human explants with 2 mmol/L gentamicin for 48 hours resulted in almost complete loss of hair cells across the entire macula. At 2 days after the end of treatment, confocal imaging showed fragments of myosin VIIa–labeled material suggestive of hair cell debris and pyknotic nuclei within the body of the epithelium, indicating apoptosis of hair cells (Fig. 8A). A few apparently intact hair cells remained, almost all of which contained an actin-labeled cytocaud-like structure (Fig. 8A, B). Supporting cells effectively closed lesions at the apical surface; at 7 days after treatment, thin actin bands at the junctions in characteristic “scar” formations crossed the spaces once occupied by hair cells (Fig. 8C); these were generated at the junctions between supporting cells where their heads had expanded to close the lesion created by the loss of the hair cell. Thin sections showed the cells to be columnar with nuclei positioned basally, just above the basement membrane, and little hair cell debris was evident by 4 days after treatment (Fig. 8D). The apical surfaces of the maculae were formed of a pavement of cells covered with microvilli distinct from the surfaces of the surrounding cells of the nonsensory tissue. This morphology was maintained for ≥26 days after treatment (Fig. 8E), the longest time period examined. The appearance of the gentamicin treated tissue was similar to that from elderly patients in which there was extensive hair cell loss at the time of surgery, as described (Fig. 2H, I).

### 3.8 Supporting cell characteristics

**Fig. 9:**
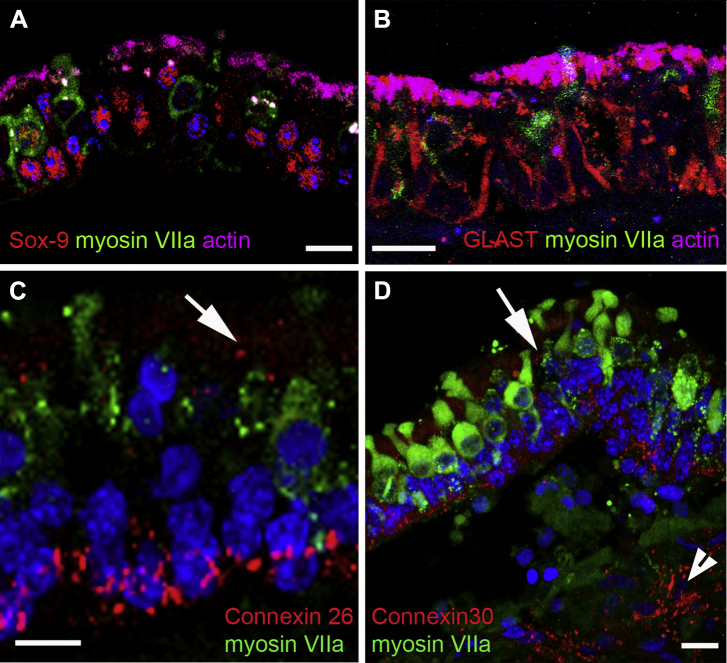
Supporting cell characteristics. (A) Labeling for Sox-9 (red) almost exclusively confined to nuclei (counterstained with DAPI, blue) at the level of those of supporting cells. Nuclei in hair cells (labeled for myosin VIIa, green) do not usually label for Sox-9, but an occasional nucleus in a myosin VIIa–labeled cell showed weak labeling for Sox-9. (Actin labeling in magenta.) (B) Labeling for l-glutamate/l-aspartate transporter (GLAST) delineates lateral membranes of supporting cells, extending down to the level of the basement membrane. (C, D) Labeling for connexins. Both CX26 (red in [C]) and CX30 (red in [D]) are present in large plaques around the cell body regions of supporting cells. Smaller labeled puncta are also present in the region close to the junctional complexes at the luminal end of supporting cells (arrows). Scale bars: (A–D) 10 μm.

To determine labels that could be used to identify supporting cells in the human vestibular system, the expression of several proteins known to be expressed in nonhuman mammalian supporting cells was examined. In mice, sox-9, 1 of several Sox family transcription factors, is expressed in the inner ear during development and in later stages becomes restricted to supporting cells. In cryosections of human utricular epithelium, sox-9 expression was found to be restricted to the nuclei of cells located in the basal region of the epithelium directly above the basement membrane (Fig. 9A). Similar labeling was also observed in sections of mouse vestibular epithelia. Supporting cells in the organ of Corti express the potassium transporter proteins KCC4 and Kir 4.1 and the glutamate transporter GLAST (Boettger et al., 2002; Taylor et al., 2012). In the human utricle, supporting cells expressed GLAST (Fig. 9B) along their plasma membranes, similar to rat vestibular epithelium (Takumi et al., 1997), but no immunolabeling for either KCC4 or Kir4.1 was detected.

Gap junctions are large and numerous between supporting cells of the sensory epithelia of the inner ear in all vertebrates examined, including humans (Bagger-Sjoback et al., 1983; Forge et al., 2003). Immunolabeling for CX and gap junction channel proteins showed both CX26 and CX30 in the human vestibular tissue, but CX43 labeling (using an antibody raised against the c-terminal of human CX43) was absent from the epithelium. Labeling for CX26 and for CX30 was extensive in the sensory epithelium, located both at the level of supporting cell bodies close to the basement membrane, and in the more apical regions close to apical tight junctions (Fig. 9C, D). Some of the labeled puncta surrounding the cell body region were quite large (Fig. 9C, D), consistent with the presence of large gap junction plaques in this region of vestibular supporting cells observed by freeze fracture (Bagger-Sjoback et al., 1983; Forge et al., 2003). Both CX26 and CX30 were also localized to cells in the mesenchyme underlying the sensory epithelium.

### 3.9 Gap junction–mediated intercellular communication

**Fig. 10:**
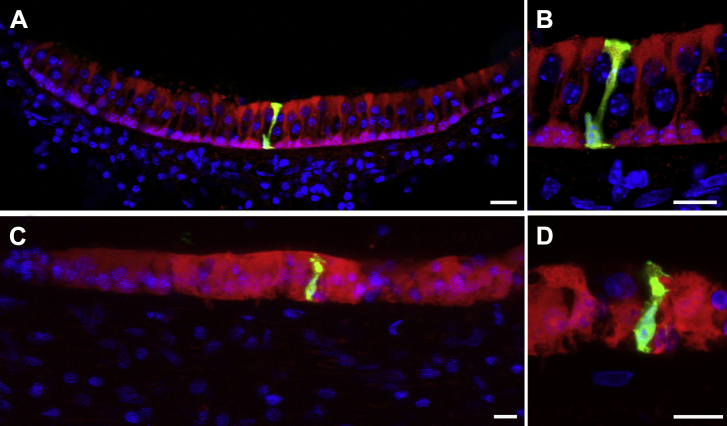
Dye transfer in viable slices of utricular maculae of a young adult (P30) mouse (A, B), and a 65-year-old human (C, D). Individual supporting cells are injected with neurobiotin (red) and fluorescein-dextran (green). Dextran is confined to the injected cell but neurobiotin spreads to all supporting cells in the slice. There is no spread to hair cells. In the human tissue, there are relatively few hair cells. Supporting cells have enlarged to occupy the spaces from which hair cells have been lost, but neurobiotin distribution shows maintenance of extensive coupling between supporting cells, and that the coupling is confined to the sensory epithelium. Data is typical of 4–5 maculae per species. Scale bars: 10 μm.

To examine the extent of gap junction–mediated intercellular communication in vestibular sensory epithelia, dye transfer studies were performed in slice preparations of viable tissue from young adult (P30) mice and humans (Fig. 10). Individual supporting cells, identified under infrared videomicroscopy both from their location and morphology, were injected via a patch pipette with neurobiotin, which can pass through gap junction channels, and fluorescein-dextran, which is too large to transfer between cells coupled by gap junctions. The fluorescein-dextran remains in the injected cell, serving to mark it and delineating its shape. In the utricular maculae of young adult mice (Fig. 10A, B), after injection into a single supporting cell, neurobiotin spread to all supporting cells across the epithelium and through the depth of the slice, but did not enter hair cells, which are known not to form gap junctions with supporting cells, nor enter the cells or extracellular spaces of the mesenchyme (Fig. 10A). Dextran was confined to the injected cell, revealing the polarized morphology of individual utricular supporting cells (Fig. 10B). The nucleus was located basally, a fine cytoplasmic phalangeal process spanned the basoapical axis of the epithelium, and a head region adhered to similar structures in adjacent supporting cells. The whole cell currents recorded during the injections indicated very low input resistance and high capacitance (data not shown), both indicators of a highly coupled syncytium (Jagger and Forge, 2006). Similar studies of human utricles in which there was significant hair cell loss produced similar electrophysiological signatures and dye transfer. While the dextran was confined to the injected cell, neurobiotin spread through the entire sensory epithelium but did not enter the remaining hair cells, the mesenchyme, or the cells of the surrounding nonsensory epithelium (Fig. 10C). This confirmed the maintenance of extensive cell coupling exclusively between supporting cells of the sensory epithelium, as well as the viability of the tissue. The extensive dye coupling revealed by the spread of neurobiotin suggests that the supporting cell population of the human vestibular system can also be considered a single large functional syncytium. The injected dyes revealed the human vestibular supporting cells also seemed to be columnar but in regions where hair cells were lost they were wider along their length (Fig. 10D), expanding into the regions once occupied by the bodies of the hair cells.

### 3.10 Apical cell junctions

**Supplementary Fig. 2:**
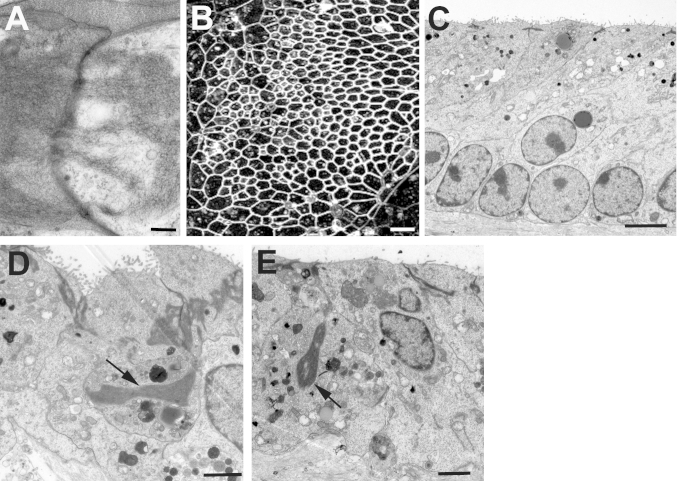
Junctional actin assemblies in mouse. (A) Rapidly frozen (by high pressure freezing), freeze-substituted tissue shows electron-dense structures in contact with the lateral membrane in the region of the adherens junction are composed of microfilaments. (B) Explanted utricular macula from a 22-month-old CBA/Ca mouse, maintained in culture for 19 days after exposure to gentamycin. All hair cells lost. Phalloidin labeling of whole mount preparation shows actin bands in the junctional region of supporting cells to be relatively thin, but more prominent than in the equivalent junctional complexes of the cells in the surrounding nonsensory epithelium. (C–E) Thin sections of utricle from a 22-month-old CBA/Ca mouse maintained in culture for 6 days after exposure to gentamicin; hair cells lost. (C) The adherens junctional region between supporting cells is simple; there is little evidence large actin assemblies. (D, E) Electron-dense structures with the appearance of junctional actin assemblies are displaced to the basal region of the cells (arrows) and detached from the plasma membrane (E). Scale bars: (A) 0.5 μm; (B) 10 μm; (C) 5 μm; (D, E) 2 μm.

**Fig. 11:**
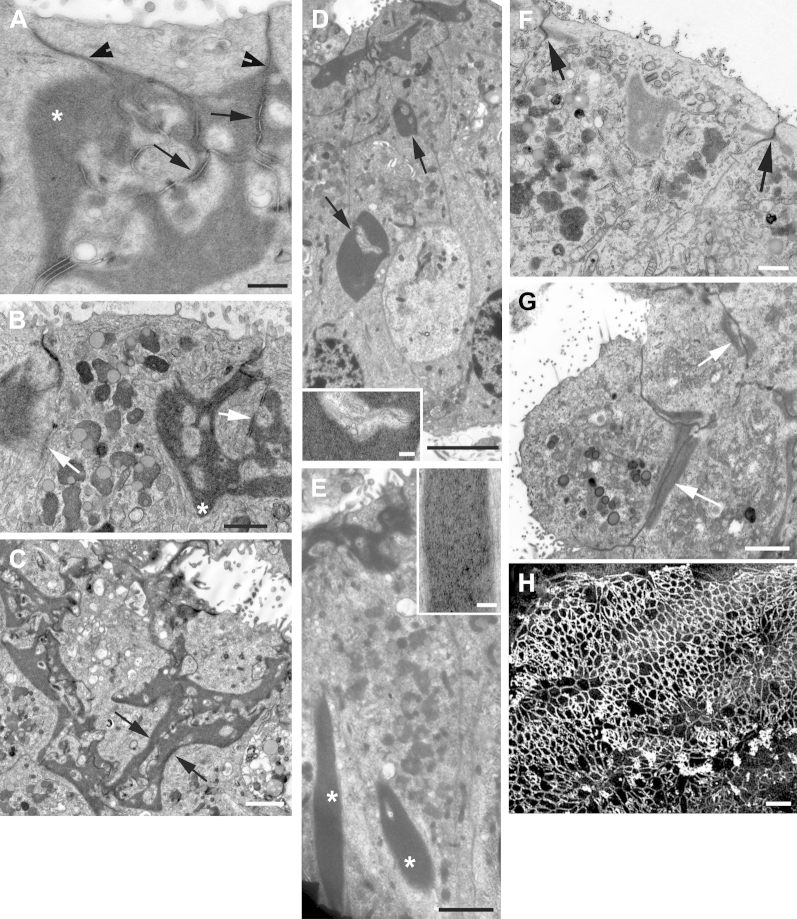
Remodeling of actin assemblies at adherens junctional complexes. (A–C) Normal morphology of junctional complexes in human utricular maculae. (A) Large, electron-dense actin structures (1 denoted by asterisk) in the region of the adherens junction contact the plasma membrane in scalloped fashion at both sides of the junction. The basal limits of the tight junctions are indicated by the arrowheads; the electron densities at the plasma membrane, which indicate the adherens junction itself, are indicated by arrows. The actin assemblies often, but not always contact the plasma membrane at the site of the adherens junction. (B) Section parallel to the long axis of the supporting cell shows the actin assembly extends quite deeply into the cells (asterisk). The arrow indicates where there is no actin assembly to appose that contacting the lateral plasma membrane in the adjacent cell. (C) Cross-section (almost parallel to the apical surface) shows the width of the actin assemblies (arrows indicate the limits at 1 intercellular junction in each of the apposed cells). (D, E) Displaced actin assemblies. In (D), arrows indicate actin assemblies in apposed cells displaced basally. The inset shows that in the lower indicated structure each actin assembly contacts the plasma membrane in the respective apposed cells. In (E), structures with an appearance identical to that of the junctional actin assemblies (asterisks) are contained within the cytoplasm of the cell body region of the supporting cell. The inset shows these structures are composed of closely packed filaments of a size consistent with that of f-actin. (F–H) Junctions in tissue after gentamicin-induced hair cell loss. (F) Seven days after the end of incubation with gentamicin. Longitudinal section shows relatively small electron densities contacting plasma membrane in the adherens junction region (arrows). An electron-dense structure similar to the junctional actin assembly is located in the center of the cell detached from the plasma membrane. (G) Four days after gentamicin exposure. Cross-section show the actin band associated with the adherens junction is thinner that in normal tissue as in panel (C). (H) Confocal image of phalloidin-labeled whole mount of tissue 19 days after gentamicin exposure reveals actin bands in the junctional region of supporting tissue to be thinner than those in “normal” tissue (e.g., Fig. 1F), although still denser and more prominent than the actin associated with the adherens junctions between cells in the nonsensory epithelium. Scale bars: (A) 0.5 μm; (B) 1 μm; (C) 2 μm; (D) 5 μm, inset 0.5 μm; (E) 2 μm, inset 0.5 μm; (F) 1 μm; (G) 2 μm; (H) 10 μm.

A further characteristic of supporting cells in mature mammalian vestibular organs is the presence of thick actin bands around the necks of the cells, to which attention has recently been drawn (Burns and Corwin, 2014; Burns et al., 2008, 2013). Labeling of the human utricular maculae with fluorophore–conjugated phalloidin confirmed recent reports (Burns et al., 2013) of wide actin rings around the necks of supporting cells in human tissue (Fig. 1E), although even tissue freshly fixed after harvesting showed areas of hair cell loss where the actin-labeled band at the junctions between supporting cells were much thinner. Fine gaps between the thick phalloidin-labeled bands indicated the space between the adjacent cells. In thin sections, dense structures were associated with the region of the adherens junctions. High-power images of thin sections of rapidly frozen, freeze-substituted mouse utricular macula (Supplementary Fig. 2A) showed these structures to be composed of closely packed cross-linked filaments indicating, along with their location, that they correspond with the actin bands revealed by phalloidin labeling. In human tissue, these actin assemblies contacted the inner surface of the plasma membrane in the adherens junction region (Fig. 11A), from just beneath the tight junction, approximately 0.5–1 μm below the apical surface, and descended some distance down the lateral plasma membrane often in a “scalloped” configuration such that several points of contact with the membrane were separated by regions from which the filamentous structures were absent (Fig. 11A, B). The points of membrane contact in 1 supporting cell sometimes directly apposed similar contacts in the supporting cell on the opposite side of the junction (Fig. 11A), but this was not always the case, even though dense bands were present along the points of contact in both apposed cells (Fig. 11B). The actin assemblies could descend deeply into the cell and the deepest extent was often within the interior of the cell away from the plasma membrane and below the level of the adherens junction to a depth sometimes of >5 μm (Fig. 11B). The width across a cell measured from images of sections cut parallel to the long axis and from sections that cut the tissue at an angle to the apical plasma membrane was ≤2 μm (Fig. 11C).

However, in some supporting cells, the electron-dense structure was essentially absent from the junctional region, even though it was present in the adjacent contacting cell (Fig. 11B). There was also evidence for the coordinated displacement of the actin bands of adjacent cells to a deeper level closer to the nuclei while still in apposition to each other on either side of the extracellular space and in contact with the plasma membranes (Fig. 11C). In addition, dense filamentous structures seemingly similar to the actin bands and formed of parallel microfilaments were present detached from the plasma membrane and entirely within the cytoplasm inside the lower regions of supporting cells (Fig. 11D). These peculiarities of actin bands were present in regions where there seemed to be significant hair cell loss and thus, may be a reflection of reorganization of supporting cells when repairing lesions caused by loss of hair cells.

As described, by 4–7 days after exposure to gentamicin, phalloidin labeling revealed typical “scar” formations at the sites of lost hair cells where the filamentous actin bands associated with the intercellular junctions were thin relative to the thick bands between adjacent supporting cells where they had not been in contact with a hair cell (Fig. 8C). Thin sections also showed junctions between adjacent supporting cells that were simple, with only a thin electron density contacting the adherens junction over a short distance, approximately 0.5 μm down the lateral membrane (Fig. 11F) and not extending deeply into the cell (Fig. 11G). In addition, where large electron-dense structures were still present in supporting cells, they were sometimes detached from the plasma membrane (Fig 11F) or found to be displaced to deeper levels of the cell. By 21 days after treatment, most phalloidin-labeled actin bands at the level of the adherens junctions between supporting cells, although thicker than those between cells in the nonsensory epithelium surrounding the macula, were thinner and less prominent than at earlier times (Fig. 11H).

Similar observations were made in explant cultures of utricular maculae from aged (22-month-old) CBA/Ca mice. At 21 days after exposure to gentamicin, phalloidin labeling revealed prominent, but relatively thin, actin bands around the necks of supporting cells (Supplementary Fig. 2B). Thin sections also showed the adherens junction-associated actin assemblies at most contact sites between supporting cells were relatively simple, but some of the larger actin assembly structures persisted (Supplementary Fig. 2C). These were sometimes displaced (Supplementary Fig. 2D) and structures resembling the junctional assemblies were also located deep in the cell detached from the plasma membrane (Supplementary Fig. 2E), similar to the situation described for human tissue. Taken together, these observations suggest that, throughout life, supporting cells in human and mouse vestibular tissues retain a capacity to reorganize their structure in response to hair cell loss and remodel their adherens junctional specializations.

## 4 Discussion

Loss of hair cells from the vestibular sensory epithelia of the inner ear is an effect of ageing. The present work with human tissue demonstrates that, in some elderly individuals, there can be almost complete loss of hair cells across the entire utricular macula, a surprisingly extensive loss much greater than that observed in the utricular maculae of mice near to the end of their normal lifespan. The present work also demonstrates the feasibility of obtaining human vestibular sensory epithelia in sufficient numbers to allow for meaningful experimental studies that could provide a means for exploring therapies for intervention to ameliorate or reverse age-related hair cell loss. An initial assessment of the harvested tissue using characteristics observed under low-power microscopy provided a degree of quality control and after this initial screening more than one-half the samples received were actually rejected, but several criteria showed that samples which passed the screening were viable and largely intact, even in samples that had arrived several days after harvesting. Samples taken at random for FM1-43 labeling showed uptake of the dye exclusively into hair cells, indicating hair cell viability; phalloidin labeling revealed the junctional patterning characteristic of the intact epithelium; DAPI labeling showed very few nuclei, and almost none at the level of supporting cells, with characteristics of apoptosis or necrosis; and dye injection into individual supporting cells revealed the integrity and viability of supporting cells. Immunolabeling of hair cells together with phalloidin and DAPI labeling also demonstrated that human vestibular tissue can be maintained ex corporeally intact for ≤4 weeks, and incubation with gentamicin led to loss of hair cells and efficient repair by supporting cells of the lesions caused, indicating retention of supporting cell activities.

However, in most samples many hair cells had lost their stereociliary bundles, most likely at the time of harvesting. The operative procedure that provides access to the inner ear (and subsequently the tumour to be removed) requires the unavoidable use of irrigation to keep bone cool during drilling to gain access to the inner ear and to remove bone dust. It is likely that, upon initial access to the vestibular system, the utricle is exposed to the irrigation washing off the otoconial membrane, which was never apparent in the harvested samples, from the apical surface of the utricular maculae and taking hair bundles with it. The loss of the hair bundles did not seem to affect viability; hair cells without bundles could survive for ≥4 weeks in their absence, but it may be a little surprising that FM1-43 entered hair cells, and that they were susceptible to gentamicin. FM1-43 is thought to enter hair cells via the transduction channels at the distal ends of the stereocilia, which may also be a route of entry for aminoglycosides (Gale et al., 2001). Although it may be that FM1-43 was labeling only a subpopulation of hair cells within the sample, the dye may also enter cells via purinergic channels at the apical surface of hair cells (Meyers et al., 2003) or via endocytosis (Griesinger et al., 2002) and some hair cells showed significant numbers of small pits reminiscent of endocytotic vesicle openings at the apical membrane (e.g., Fig. 5G). This may also provide 1 route of entry for gentamicin (Hashino and Shero, 1995). The effectiveness of the gentamicin in damaging the hair cells, illustrated by the labeled hair cell debris and the pathologic feature of the cytocauds inside all remaining hair cell bodies soon after the end of incubation with the drug, may also have been enhanced by the relatively long time of exposure to the drug, 48 hours, and relatively high concentration, 2 mmol/L, enabling a critical concentration to accumulate inside the hair cell. Nevertheless, despite the fact that hair bundles were often lost, taken all together our results confirm the potential of the excised human vestibular sensory tissues for studies of means to protect hair cells from lethal injury and of strategies for hair cell regeneration.

### 4.1 Spontaneous hair cell regeneration is retained throughout life in the human vestibular tissue

In this context, a particularly significant finding of the present study is the observation in tissue fixed immediately after removal from the patient, of hair bundles with immature characteristics, even in utricular maculae of elderly people. Although hair cells are produced throughout life in some nonmammalian vertebrates (Collado et al., 2008; Rubel et al., 2013), and in birds there seems to be a constant turnover of vestibular hair cells (regenerated ones arising to replace those that die after mitosis of supporting cells; Jorgensen and Mathiesen, 1988; Roberson et al., 1992), it has been demonstrated that there is little if any continuing vestibular hair cell production past early postnatal life in mice (Burns et al., 2012), a period that in humans would occur prenatally. (Mice are altricial animals in which the latter stages of inner ear maturation occur after birth whereas in humans the inner ear is fully mature by 3 months of gestation.) It seems, therefore, unlikely that the immature hair cells in the human utricles are the consequence of continuous hair cell production throughout life. It has also been suggested that sublethal injury to hair cells can result in loss of hair bundles and then their regrowth (bundle “repair”). This has been reported after gentamicin treatment of mature utricular maculae of mice maintained in organoptypic culture (Zheng et al., 1999), but that study did not show the presence of immature bundles and the phenomenon has not been confirmed by others, nor does it seem to occur in experimental animals in vivo (Forge et al., 1998). Bundle repair has also been reported in the saccular maculae of frogs (Gale et al., 2002), but some characteristics of that repair process, for example, the presence of cells with both mature and immature bundles on a single hair cell, were not observed in the present study. Furthermore, although loss of hair bundles with persistence of an otherwise undamaged hair cell, was common in our samples, with prolonged incubation no evidence of the reemergence of immature hair bundles on surviving “bald” hair cells was detected. It, thus, seems reasonable to conclude that the immature hair bundles denote the presence of regenerating hair cells.

The patients from whom samples were obtained had no known exposure to agents that would have caused hair cell injury, such as aminoglycoside antibiotics (indeed tissue from patients known to have received such treatment was excluded from the study), but loss of hair cells was evident in most samples either through the relatively low density and wide separation of hair cells and/or the presence of “scar” patterns typically formed when supporting cells close lesions created by loss of hair cells. In addition, in tissue from some of the oldest patients, there were very few hair cells. Thus, the apparent regeneration was most likely triggered in response to an age-related hair cell loss and could occur even when such loss was extensive. These results then suggests that, as in other mammals, the vestibular sensory epithelia in humans may retain a limited, low-level capacity for spontaneous hair cell regeneration, and that this capability is maintained throughout life. The observation of immature hair bundles in the utricular maculae of aged CBA/Ca would support this contention.

Regenerating hair cells in the vestibular organs of guinea pigs and mice are thought to arise through direct, nonmitotic transdifferentiation of supporting cells into hair cells (Li and Forge, 1997; Lin et al., 2011). Recent work has suggested possibilities for enhancing this phenotypic conversion, either through manipulation of the Notch-Delta lateral inhibition pathway that regulates hair cell-supporting cell fate during embryonic development (Lin et al., 2011) or via transfection of supporting cells with the gene encoding the transcription factor Atoh-1 (Kawamoto et al., 2003; Shou et al., 2003; Staecker et al., 2007) that is considered to be necessary and sufficient to determine differentiation of a precursor as a hair cell during development (Bermingham et al., 1999; Woods et al., 2004). Our results indicate that the environment of the vestibular sensory epithelium in humans could be responsive to such guidance and open the way for testing these manipulations in human tissue.

### 4.2 Stereociliary pathologies may be an age-related effect

Many hair cells in the utricular maculae presented with pathologic characteristics, such as fused, elongated, and internalized stereocilia. The tissue was obtained from patients with a lesion on the vestibular nerve and most sections of the tissues revealed the presence of striated “Luse bodies,” which consist of regularly arranged collagen bundles, in the mesenchyme underlying the sensory epithelium. Luse bodies have been described as a pathologic feature associated with peripheral nerve disorders, including vestibular schwannomas (Friedmann et al., 1965). However, other than the stereociliary abnormalities, there was no evidence of pathologic effects in the epithelium, either the hair cell or supporting cells, and it has been reported that destruction of Scarpa's ganglion, which contains the cell bodies of the nerves that innervate the vestibular end-organs, does not have obvious effects upon the sensory epithelia (Suzukawa et al., 2005). It is thus likely that the growth of the schwannoma that created the need for the surgery was not a cause of the stereociliary pathologies.

Fusion of stereocilia, the formation of giant stereocilia and apparent internalization of stereocilia, have all been observed in the organ of Corti in optimally preserved preparations of human temporal bones from elderly individuals and have been ascribed to the effects of ageing (Wright, 1981; Wright et al., 1987). We have also observed similar features in the organs of Corti of aged CBA/Ca mice (Taylor and Forge, unpublished data), where the internalization of stereocilia corresponded with evidence of autophagy in hair cells, as well as in the utricular maculae of the aged animals as reported here. Thus, the stereociliary anomalies we observed most likely derive from effects of ageing (we did not have a sufficiently large numbers of samples across age groups, especially younger ones—<50 years—to perform a detailed study of the progressive effects of ageing). We have reported recently that, in mice deficient in the actin bundling protein plastin 1, there is a thinning of stereocilia most notably at their distal ends (Taylor et al., 2014), an abnormality seen in the present study. This was associated with a late (young adult) onset, progressive hearing loss suggesting a possible relationship of the stereociliary anomalies to age-related hearing loss. Defects in espin also cause thinning of stereocilia (Sekerkova et al., 2011), and mutations in the gene encoding myosin VI, result in detachment of the apical membrane of the hair cell from the cuticular plate in the regions between the proximal tapers of the stereocilia, and fusion of stereocilia, a phenomenon also observed here in the human vestibular hair cells. It seems likely that “stereociliopathies” of the kind observed here might result from a failure in the proper maintenance of stereocilia and be an early and persisting effect of ageing on hair cells. Hair cells with fused or elongated stereocilia would be unable to function effectively, if at all, and thus vestibular (and likely auditory) functional deficits resulting from effects on stereocilia may precede for some time any overt hair cell loss during ageing.

### 4.3 Supporting cell characteristics

For studies aimed toward regenerating hair cells, it is of importance to identify markers for supporting cells, the source of regenerated hair cells and the present work indicates that this cell population retains a number of defining characteristics throughout life, even after the extensive hair cell loss that occurs with ageing. In a preliminary transcriptome analysis of the human vestibular tissue (performed in collaboration with Prof Mike Lovett), the transcript of the transcription factor sox-9 was identified at high levels. Sox-9 has been implicated in differentiation of cells in the developing sensory epithelia of the inner ear of mice and becomes restricted to supporting cells during embryonic life. We have now identified sox-9 as a useful label for supporting cells in the mature human tissue; antibodies to sox-9 provided almost exclusive labeling of supporting cell nuclei in both the utricle and cristae. Its retention into adulthood has not previously been reported, and the role it might play in the mature tissue is not known.

We also sought the presence of markers of the specialized functional activity of supporting cells, which we have identified in previous work on supporting cells of the organ of Corti of mice (Taylor et al., 2012): the K^+^ transporter KCC4, the inwardly rectifying K^+^ channel Kir4.1 (KCNJ10), the glutamate transporter GLAST, and CX, which form subunits of gap junction channels. We did not detect KCC4 or Kir 4.1, which has been shown to be expressed at the nerve calyx but not by supporting cells in mouse tissue (Udagawa et al., 2012). Although the inability to detect Kir4.1 in the human tissue might be because of the susceptibility of the calyx ending to degeneration upon explantation, the absence of these potassium transport proteins from the supporting cell membranes in the vestibular sensory epithelia points to a difference in the mechanisms of potassium handling between vestibular and cochlear supporting cells. However, antibodies to GLAST provided robust labeling of supporting cell membranes, even in regions of significant hair cell loss suggesting that the specializations of supporting cells persist even when hair cells and local innervation have disappeared. There was also robust labeling for both CX26 and CX30 in the sensory epithelium. Mutations in the gene encoding CX26 are the most common cause of inherited hearing loss, but patients with CX26-associated deafness do not show a vestibular phenotype (Jagger and Forge, 2014). It would seem, therefore, that the presence of CX30 can compensate for defects in CX26 in the vestibular system, but not in the cochlea.

In the organ of Corti, gap junctions provide for extensive coupling between supporting cells such that supporting cell populations fall into 2 separate large functional syncitia, one medially around inner hair cells and one laterally around the outer hair cells along the entire length of the sensory epithelium (Jagger and Forge, 2006). Supporting cells in the basilar papilla of birds are similarly extensively coupled but apparently in only a single compartment (Jagger et al., 2014). The results of the dye coupling studies in the present work now show the supporting cells of the mammalian vestibular sensory epithelia are similarly extensively coupled. The coupling was confined exclusively to the sensory epithelium seemingly creating a single large compartment encompassing the entire sensory epithelium but separated from the tissues surrounding and underlying it. In the human tissues examined, there was significant, presumably age-related hair cell loss evidenced by the relatively low density of hair cells in comparison with the tissue from the young mice, but this did not seem to affect the extent of supporting cell coupling. In the organ of Corti of mice, CX30 is the predominant CX in the gap junctions between the supporting cells that surround the outer hair cells, and in a strain of mouse from which CX30 has been ablated, the pattern of lesion repair by supporting cells when hair cells die is disrupted (Forge et al., 2013). In that model, supporting cells fail to expand to fill the spaces left by dying hair cells. Supporting cell expansion is an essential phase of epithelial repair in the inner ear, and seems to rely on functional coupling via gap junctions (Jagger et al., 2014). Consequently, the maintenance of coupling with extensive hair cell loss as observed in this present study is likely to be important in enabling coordination of repair responses of supporting cells as hair cells progressively die, and ensuring maintenance of the tissue's integrity throughout human life.

### 4.4 Remodeling of adherens junctions between supporting cells

It has been suggested that in mammals the actin assemblies associated with the adherens junctions at the necks of supporting cells provide enhanced mechanical stability of the epithelium, and may be related to the inability of supporting cells in the mammalian inner ear to undergo cell division (Burns and Corwin, 2014; Burns et al., 2013). A careful examination of these structures in the present study suggests they become remodeled after hair cell loss. As this previous work has suggested, normally in the human vestibular sensory epithelia the actin assemblies associated with adherens junctions are unusually wide and deep. However, in regions of hair cell loss in tissue fixed immediately upon arrival in the laboratory and even more so in tissue from which all hair cell had been ablated after exposure to gentamicin, these actin assemblies were often much less extensive. Although it has been known for some time that the new intercellular junctions that form between supporting cells when closing the lesion caused by loss of a hair cell initially have little actin (Forge, 1985; Li et al., 1995; Meiteles and Raphael, 1994; Raphael and Altschuler, 1991), our present results indicate that existing wide junctions can become remodeled. It seemed that entire actin assemblies could be released from the membrane and displaced deeply into the cell, or sometimes were displaced while still attached to the membrane and to become released from the membrane into the cytoplasm of cell body region of the cell, whereas at the junctional region reformation of smaller actin assemblies occurred. What the eventual fate of these detached actin assemblies might be was not clearly resolved, but presumably they are broken down. Nor was the mechanism that underlies displacement down the membrane clear. Nevertheless, the result was that, after hair cell loss, the junctions between supporting cells were much less complex than in undamaged regions of tissue with a complement of hair cells. This clearly indicates that supporting cells in the human vestibular sensory epithelia retain a capacity to undergo structural remodeling in response to changing environmental conditions. It may also have implications for attempts to replace lost hair cells using exogenously applied precursors derived from stem cells. The incorporation of such cells into the epithelium requires disruption of the junctions between supporting cells. Disruption of the extensive junctional complexes normally present in the epithelium would be difficult to achieve, but it may be much less so in epithelia where hair cell loss has occurred and the junctions are less complex.

This study demonstrates not only the viability of human vestibular epithelia harvested during trans-labyrinthine surgery, but also characterizes some effects of ageing. It demonstrates that tissue harvested from the human inner ear provides an in vitro model that can be used in translational studies for regenerative therapeutics and for studies of ageing.

## Disclosure statement

The authors have no conflicts of interest to disclose
